# Explainable breast cancer molecular expression prediction using multi-task deep-learning based on 3D whole breast ultrasound

**DOI:** 10.1186/s13244-024-01810-9

**Published:** 2024-09-19

**Authors:** Zengan Huang, Xin Zhang, Yan Ju, Ge Zhang, Wanying Chang, Hongping Song, Yi Gao

**Affiliations:** 1 School of Biomedical Engineering, Shenzhen University Medical School, Shenzhen University, Shenzhen, Guangdong 518055 China; 2 Department of Ultrasound, Xijing Hospital, Fourth Military Medical University, No. 127 Changle West Road, Xi’an, 710032 China

**Keywords:** Breast cancer, Deep learning, Ultrasound imaging

## Abstract

**Objectives:**

To noninvasively estimate three breast cancer biomarkers, estrogen receptor (ER), progesterone receptor (PR), and human epidermal growth factor receptor 2 (HER2) and enhance performance and interpretability via multi-task deep learning.

**Methods:**

The study included 388 breast cancer patients who received the 3D whole breast ultrasound system (3DWBUS) examinations at Xijing Hospital between October 2020 and September 2021. Two predictive models, a single-task and a multi-task, were developed; the former predicts biomarker expression, while the latter combines tumor segmentation with biomarker prediction to enhance interpretability. Performance evaluation included individual and overall prediction metrics, and Delong’s test was used for performance comparison. The models’ attention regions were visualized using Grad-CAM + + technology.

**Results:**

All patients were randomly split into a training set (*n* = 240, 62%), a validation set (*n* = 60, 15%), and a test set (*n* = 88, 23%). In the individual evaluation of ER, PR, and HER2 expression prediction, the single-task and multi-task models achieved respective AUCs of 0.809 and 0.735 for ER, 0.688 and 0.767 for PR, and 0.626 and 0.697 for HER2, as observed in the test set. In the overall evaluation, the multi-task model demonstrated superior performance in the test set, achieving a higher macro AUC of 0.733, in contrast to 0.708 for the single-task model. The Grad-CAM + + method revealed that the multi-task model exhibited a stronger focus on diseased tissue areas, improving the interpretability of how the model worked.

**Conclusion:**

Both models demonstrated impressive performance, with the multi-task model excelling in accuracy and offering improved interpretability on noninvasive 3DWBUS images using Grad-CAM + + technology.

**Critical relevance statement:**

The multi-task deep learning model exhibits effective prediction for breast cancer biomarkers, offering direct biomarker identification and improved clinical interpretability, potentially boosting the efficiency of targeted drug screening.

**Key Points:**

- *Tumoral biomarkers are paramount for determining breast cancer treatment*.
- *The multi-task model can improve prediction performance, and improve interpretability in clinical practice*.
- *The 3D whole breast ultrasound system-based deep learning models excelled in predicting breast cancer biomarkers*.

**Graphical Abstract:**

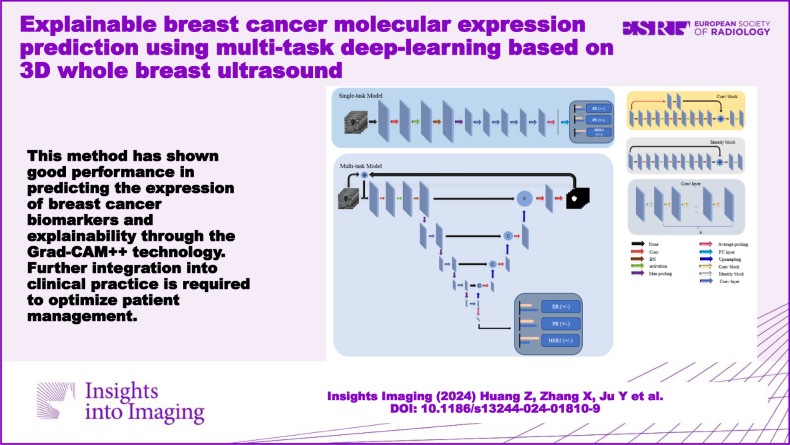

## Introduction

Breast cancer is a prevalent form of cancer among women and contributes substantially to cancer-related fatalities [1]. Breast cancer could be classified based on the expression of these tumoral biomarkers: estrogen receptor (ER), progesterone receptor (PR), and human epidermal growth factor receptor 2 (HER2) [2]. These biomarkers are crucial in clinical decision-making, as they assist in predicting the response to specific therapies and offering valuable prognostic insights [3].

In recent years, medical image analysis, such as lesion detection, diagnosis, and disease monitoring, has significantly advanced through the use of deep learning-based artificial intelligence (AI) techniques [4–6]. Several studies have demonstrated that AI techniques can identify breast cancer biomarkers and molecular subtypes from hematoxylin-eosin-stained breast cancer pathological images [3, 7]. However, noninvasive imaging is a more patient-friendly option than histopathological examination. Some studies have utilized deep learning to determine biomarkers based on multi-modal ultrasound (US) imaging [8, 9]. Utilizing US-based assessment to identify molecular subtypes can significantly enhance the accuracy of breast cancer diagnosis. This approach offers a deeper insight into the disease’s heterogeneous nature while minimizing patient discomfort. Such noninvasive techniques are pivotal in understanding the complexity of breast cancer without adding unnecessary patient strain. Though promising, these studies suffer from a variety of shortcomings. Multi-modal US imaging is difficult to standardize as it heavily relies on operator expertise [10]. In response, some researchers have explored using breast Magnetic Resonance Imaging (MRI) as an alternative to US. However, breast MRI is often not available at the initial point of cancer detection, and its use is not universally standard across different countries for all breast cancer patients [11]. While MRI has many advantages, its drawbacks also limit its wide adoption, especially in resource-limited areas. Furthermore, while some studies [3, 8] claimed their methods were “interpretable,” their AI models lack explicit constraints but directly provide final predictions. This lack of constraints renders their results unexplainable in most scenarios [3].

To address these shortcomings, a three-dimensional whole-breast US system (3DWBUS) has been recommended. 3DWBUS integrates automation and 3D scanning technology into a system that embodies efficiency, reproducibility, and comprehensive tumor analysis [12, 13]. In addition, 3DWBUS has been shown to increase the early detection rate of breast cancer among women with dense breast tissue [14]. The remaining challenge is creating more explainable AI technology. The multi-task learning neural network has been shown to work well under the constraint of tumor segmentation [15], improving the “black-box” deep learning model’s transparency [16].

This study assessed the effectiveness of single-task and multi-task learning approaches for predicting three clinically relevant breast cancer biomarkers using 3DWBUS. We hypothesize that incorporating a segmentation task to the current approach could improve the accuracy of predicting biomarker expression. This enhancement could also increase interpretability through advanced model visual explanation technologies, like Grad-CAM + + [17].

## Materials and methods

### Study population

**Fig. 1:**
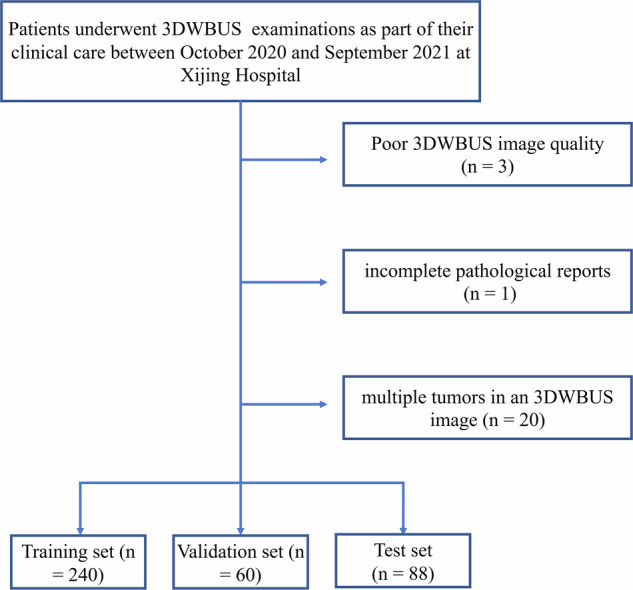
Flow chart of patient recruitment

Patients who consecutively underwent 3DWBUS examinations at Xijing Hospital from October 2020 to September 2021, as part of their routine clinical care, were included in this study. All patients met the following inclusion criteria: (a) had biopsy-proven breast cancers; (b) pre-biopsy images on 3DWBUS and therefore these images do not have biopsy scars. The exclusion criteria were as follows: (a) poor 3DWBUS image quality (*n* = 3); (b) incomplete pathological reports (*n* = 1); (c) multiple tumors in a 3DWBUS image (*n* = 20). Figure 1 shows a flow chart of patients’ recruitment.

In this study, we did not record the breast density and all are mass-type lesion cases and invasive ductal carcinoma cases.

### 3DWBUS images acquisition

All 3DWBUS images used in this study were acquired using Invenia 3DWBUS 2.0 (GE Healthcare). Acquisition parameters are reported in Table 1.

**Table 1:** Acquisition parameters of the clinically acquired 3DWBUS

Orientation	Left, Right
Matrix (pixels)	841 × 482
Slice thickness (mm)	0.48
Transducer frequency range (MHz)	6–15
Soft Tissue Thermal Index	0.25
Mains frequency	50/60
Number of piezoelectric elements	768

### Model construction

#### Tumor annotation and image preprocessing

One radiologist manually delineated the tumor on each section using open-source medical imaging software (3D Slicer [18, 19], version 5.0.3; https://www.slicer.org/). An experienced radiologist then manually reviewed and corrected the boundaries of the segmented volumes of interest. Importantly, both radiologists conducted their analyses blinded to any clinical and pathological information. Considering the multi-view scan of 3DWBUS scans, the radiologist exclusively reviewed the volume that presents the lesion in the clearest view.

Data preprocessing included cropping, resizing, intensity clipping, and normalization. After preprocessing, each image was resized into 128 × 128 × 128 and normalized to 0–1. The details of data preprocessing are described in “Data preprocessing” in Supplementary Materials.

#### Single-task model architecture’

**Fig. 2:**
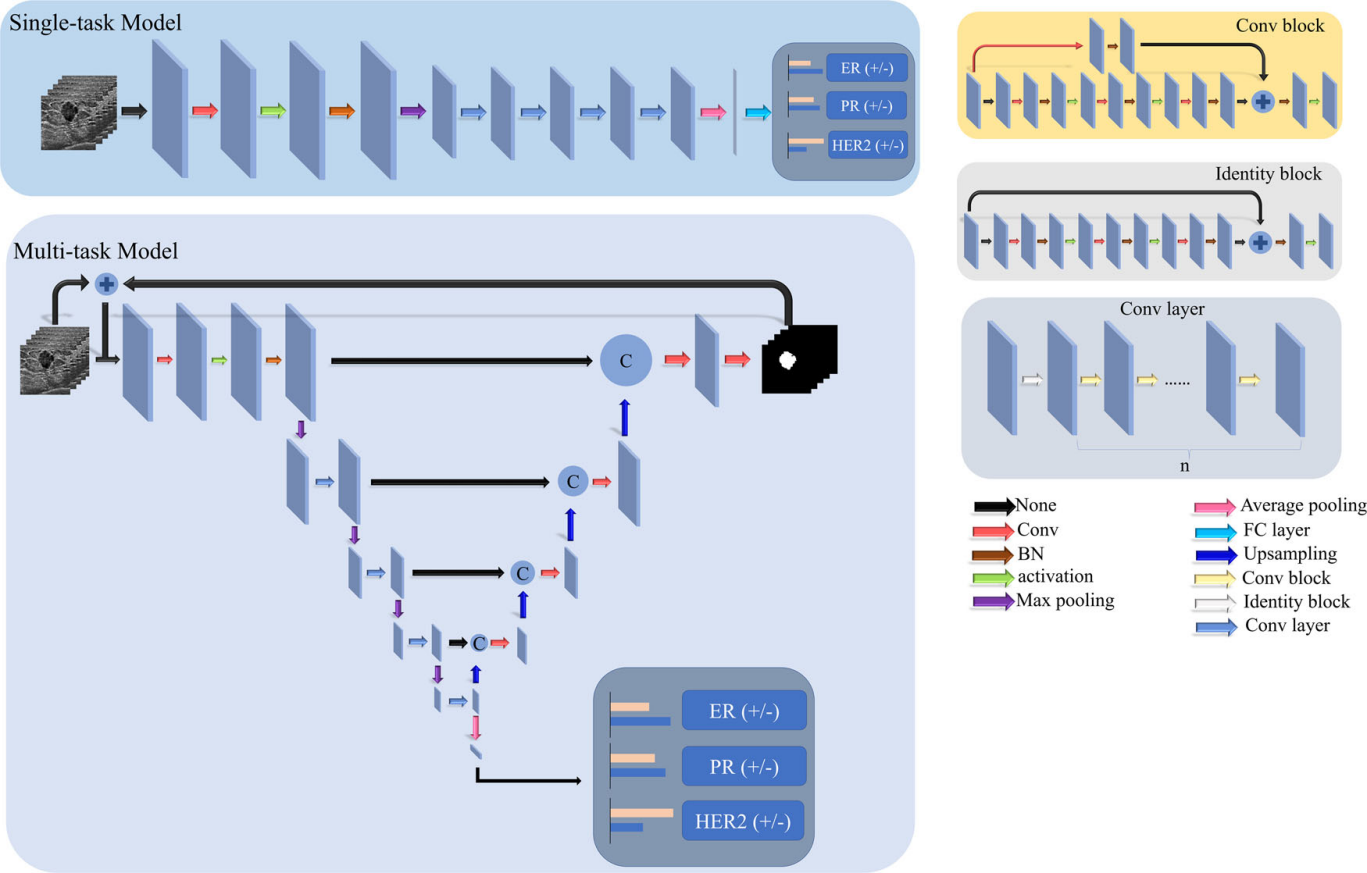
Neural network architecture (Conv, Convolutional Layer; BN, batch normalization layer; FC, fully connected layer)

A modified 3D ResNet model [20] was employed in the single-task setting to analyze 3DWBUS data. The architecture of this model is illustrated in Fig. 2 (top panel). The 3DWBUS images first underwent several layers, including a convolutional layer, batch normalization layer, and rectified linear unit activation layer, followed by a maximum pooling layer. Then four modules, each with a 3D convolutional block and multiple 3D identity blocks, were applied to the images. Finally, an average pooling layer transformed the feature map into a feature vector. A fully connected layer with a sigmoid activation generated the prediction probability.

#### Multi-task model architecture

The model architecture is shown in Fig. 2 (bottom panel). Inspired by Zhou et al [21], we employed the same ResNet architecture used in the single-task model for the multi-task model, adding an extra max-pooling operation for feature extraction and classification. Then a segmentation branch consisting of convolutional and upsampling blocks attempted to recover the segmentation results. The upsampling blocks incorporated skip connections with the corresponding feature map from the encoding stage to capture more contextual information.

In the multi-task framework, a 3DWBUS volume is input into the network, yielding a 3D segmentation probability map and a classification score. During training, this input volume is enhanced by an element-wise addition with the segmentation probability map from the previous iteration. This modulation technique aims to sharpen the network’s focus on tumor regions, thereby improving classification performance and explanatory capability.

#### Model training and implementation

The training set was used to develop the model, the validation set was utilized to select hyperparameters, and the test set was used to evaluate the models. The single-task and multi-task learning models were trained for 250 epochs with a batch size of 8 using the adaptive moment estimation optimizer with a learning rate of 10e–4. We used the cross-entropy loss for the classification task and the dice loss for the segmentation task to train the models. To address the potential overfitting issue, we adopted data augmentation techniques, including random rotation, flipping, and translation.

#### Model visualization

We utilized Grad-CAM + + to investigate how the neural network identified pertinent tumor information and whether the segmentation task constraint enhanced its interpretability. Following the original Grad-CAM + + setting, we selected deep feature maps from the final convolutional layer. This layer encapsulates the network’s most abstract and informative features, offering a balance between high-level semantics and spatial detail, allowing us to analyze the focus of both single-task and multi-task models. Subsequently, we computed the gradients between the prediction results and feature maps utilizing Grad-CAM + +. This process generated class-specific heatmaps highlighting the image regions influencing the network’s predictions, providing visual attention visualization. The heatmaps were color-coded using the turbo color scheme, with blue or red indicating higher predictive value and purple representing lower predictive value. Analyzing these heatmaps provided insights into the neural network’s decision-making process concerning relevant tumor information, where higher predictive values indicated a greater contribution to its decisions.

Moreover, t-distributed stochastic neighbor embedding (t-SNE) algorithm [22] was employed to reduce the 2048-dimensional features extracted by the multi-task and single-task models to three dimensions in the test set. Subsequently, these reduced features were visualized in three-dimensional space to illustrate the differences in feature distribution between samples with positive or negative expressions for ER/PR/HER2. For the sake of transparency and to facilitate reproducibility, we have fixed the relevant parameters of t-SNE as follows: perplexity set to 30, learning rate at 80, number of iterations at 1000, the initial method chosen as ‘PCA’, and a minimum gradient norm (min_grad_norm) of 1e-7.

#### Statistical analysis

We performed statistical analyses using R (version 4.0.4; http://www.r-project.org/) and MedCalc (version 15.8; http://www.medcalc.org/).

To investigate whether the sample size in our study was sufficient to detect an area under the receiver operator characteristic curve (AUC) value different from 0.500, we estimated the sample size based on the following parameters: power, 80%; two-sided significance level, 0.05; the alternative hypothesis of the true AUC values of multi-task model for each biomarker’s prediction in test set compared with the null hypothesis of AUC = 0.5; the ratio of classes, the real ratios in our study (ER = 20 negative/68 positive cases, PR = 26 negative/62 positive cases, HER2 = 38 negative/50 positive cases) [23].

We evaluated the model performance in two ways: individual and overall. For individual evaluation, we calculated AUC with 95% confidence intervals (CI) to evaluate the predictive performance of the different models for individual biomarkers. Then we calculated accuracy (ACC), sensitivity (SEN), and specificity (SPEC) from the receiver operating characteristic (ROC) curve based on the optimal cut-off value obtained by maximizing the Youden index from the ROC curve analysis. Additionally, we used DeLong’s test to compare the performance of different models, considering *p* values less than 0.05 to be statistically significant.

Furthermore, as we conducted a model for predicting multiple biomarkers, which can be considered a multi-label task model, we computed several metrics to compare the overall performance of different models. These metrics included macro ACC, macro Recall, macro Precision, macro F1 score, and macro AUC, drawing inspiration from existing research in multi-label classification [24].

## Results

### Population and scan parameter description

Finally, a total of 388 3DWBUS images of breast cancer were collected. The breast cancer cases were classified into three molecular subtypes using immunohistochemical findings and silver-enhanced in situ hybridization test, based on ER, PR, and HER2 information. Among the collected images, 300 cases expressed positive PR, 276 expressed positive ER, and 176 expressed positive HER2.

The cohort was randomly divided at the patient level into a training set (*n* = 240, 62%), a validation set (*n* = 60, 15%), and a test set (*n* = 88, 23%). The characteristics of the breast cancer cases used in the study are listed in Table 2. The tumor characteristics of the training, validation, and test sets were not significantly different (all *p* values > 0.05).

**Table 2:** Baseline characteristics

Characteristic	All patients (*n* = 388)	Training and validation sets (*n* = 300)	Test set (*n* = 88)	p*
Age (years)	52 ± 12	52 ± 13	54 ± 10	0.06
BMI (kg/m^2^)	23.4 ± 3.0	22.3 ± 2.9	23.6 ± 3.1	0.85
Family history, *n* (%)	27 (7)	21 (7)	6 (7)	0.94
Memopause, *n* (%)	204 (53)	153 (51)	51 (58)	0.26
ER				0.87
positive	300	232	68	
negative	88	68	20	
PR				0.99
positive	276	214	62	
negative	112	86	26	
HER2				0.62
positive	160	122	38	
negative	228	178	50	

p* values indicated comparisons of the difference between different datasets

*N* represented the number of involved patients in each dataset

### Individual prediction performance of different models

**Fig. 3:**
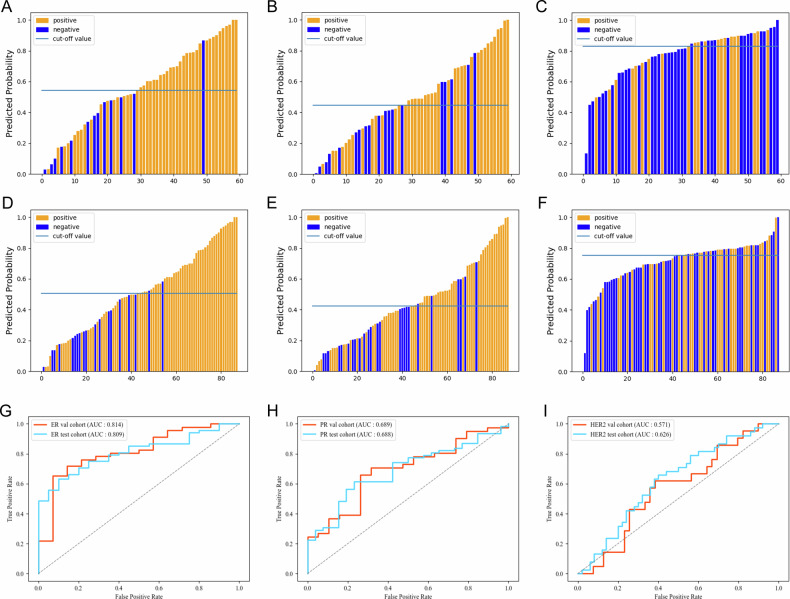
Predicted probability of the single-task model for each breast biomarker in validation set (**A**–**C**) and test set (**D**–**F**). The prediction performances of the single-task model in the validation set and test set for each breast biomarker (**G**–**I**)

**Fig. 4:**
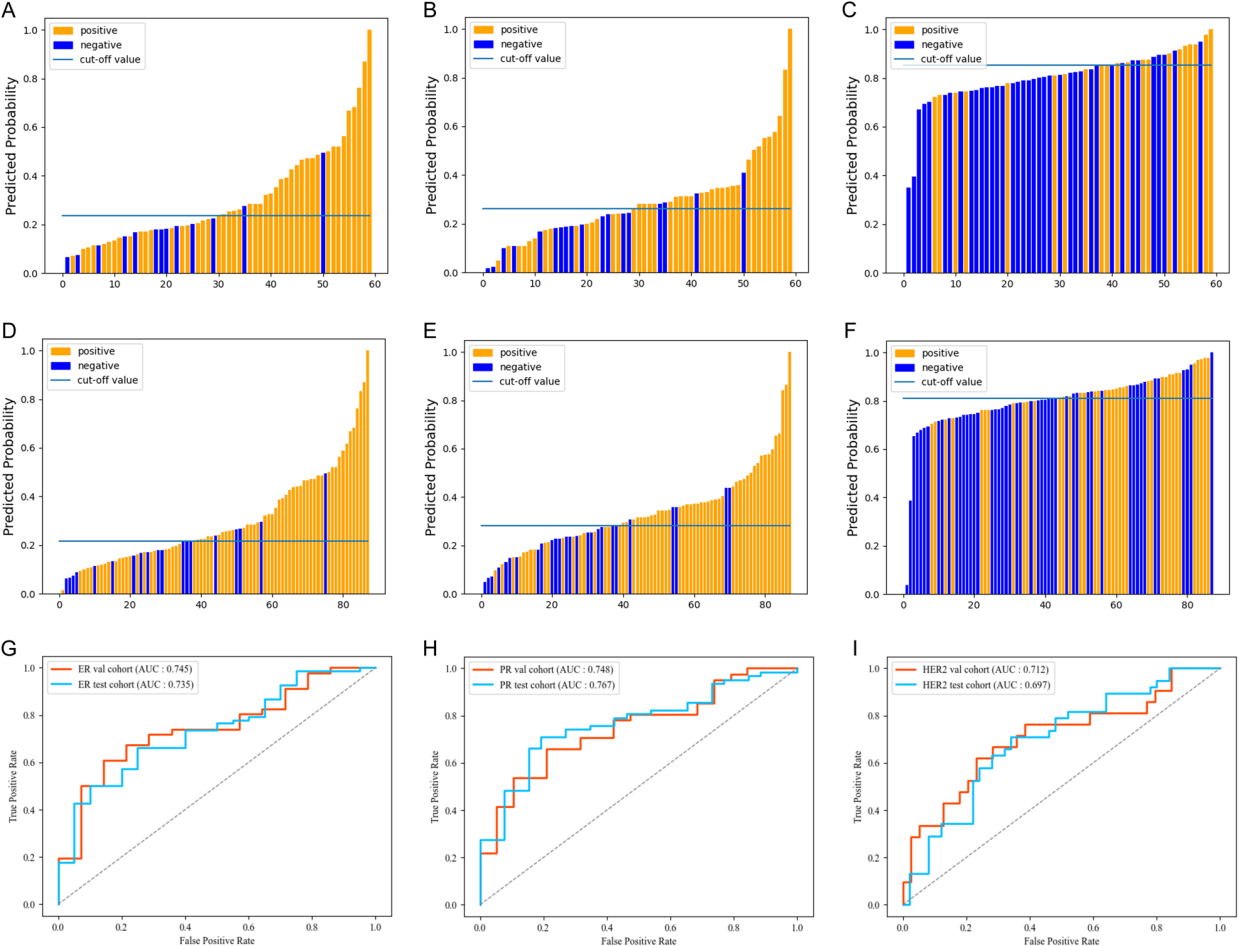
Predicted probability of the multi-task model for each breast biomarker in validation set (**A**–**C**) and test set (**D**–**F**). The prediction performances of the multi-task model in the validation set and test set for each breast biomarker (**G**–**I**)

The performance of the individual prediction for each biomarker of the different models is shown in Table 3. The predicted probability of our single-task and multi-task models for each breast biomarker in training and test sets is shown in Figs. 3a–f and 4a–f.

**Table 3:** Prediction performance of single-task model, multi-task model for individual biomarker

	AUC (95% CI)	ACC (95% CI)	SEN (95% CI)	SPEC (95% CI)	p*
**Validation set (*n*** = **60)**					
ER					
Multi-task model	0.745 (0.605–0.885)	0.667 (0.548–0.786)	0.609 (0.486–0.732)	0.857 (0.768–0.946)	0.446
Single-task model	0.814 (0.686–0.941)	0.717 (0.603–0.831)	0.652 (0.531–0.772)	0.929 (0.864–0.994)	
PR					
Multi-task model	0.748 (0.621–0.876)	0.700 (0.584–0.816)	0.659 (0.539–0.779)	0.789 (0.686–0.892)	0.481
Single-task model	0.689 (0.548–0.831)	0.683 (0.565–0.801)	0.659 (0.539–0.779)	0.637 (0.515–0.759)	
HER2					
Multi-task model	0.712 (0.562–0.855)	0.717 (0.603–0.831)	0.619 (0.496–0.742)	0.769 (0.662–0.876)	0.182
Single-task model	0.571 (0.421–0.722)	0.617 (0.494–0.740)	0.619 (0.496–0.742)	0.615 (0.492–0.738)	
**Test set (*n*** = **88)**					
ER					
Multi-task model	0.735 (0.618–0.853)	0.682 (0.585–0.779)	0.662 (0.563–0.761)	0.750 (0.660–0.840)	0.295
Single-task model	0.809 (0.717–0.900)	0.693 (0.597–0.789)	0.632 (0.531–0.733)	0.900 (0.837–0.963)	
PR					
Multi-task model	0.767 (0.664–0.870)	0.739 (0.647–0.831)	0.710 (0.615–0.805)	0.808 (0.726–0.890)	0.248
Single-task model	0.688 (0.573–0.803)	0.659 (0.560–0.758)	0.613 (0.511–0.715)	0.769 (0.681–0.857)	
HER2					
Multi-task model	0.697 (0.586–0.808)	0.682 (0.585–0.779)	0.711 (0.616–0.806)	0.600 (0.498–0.702)	0.394
Single-task model	0.626 (0.509–0.744)	0.625 (0.524–0.726)	0.658 (0.559–0.757)	0.660 (0.561–0.759)	

p* values indicated comparisons of DeLong’s test between different single-task model and multi-task model

*N* represented the number of involved patients in each dataset

The single-task model achieved an AUC of 0.814 in the validation set and an AUC of 0.809 in the test set for ER prediction, an AUC of 0.689 in the validation set and 0.688 in the test set for PR prediction, and predicted HER2 with an AUC of 0.571 in the validation set and AUC of 0.626 in the test set (all *p* < 0.001 for the AUC; Fig. 3g–i). According to DeLong’s test, there were no significant differences in the Receiver Operating Characteristic (ROC) curves between the validation and test sets across all biomarker predictions (all *p* values > 0.05). This suggests that the model did not exhibit overfitting.

The multi-task model with an AUC of 0.745 in the validation set and an AUC of 0.735 in the test set for ER prediction, an AUC of 0.748 in the validation set and an AUC of 0.767 in the test set for PR prediction and predicted HER2 with an AUC of 0.712 in the validation set and AUC of 0.697 in the test set (all *p* < 0.001; Fig. 4g–i). According to DeLong’s test, there was still no significant difference in ROC curves between the validation and test set for each biomarker prediction (all *p* > 0.05), also indicating no overfitting.

Furthermore, DeLong’s test was utilized to compare the ROC curves of different models pairwise, and the results are presented in Table 3. Regarding ER prediction, the single-task model showed better diagnostic performance than the multi-task model both in the validation set (AUC = 0.814 vs. 0.745, *p* = 0.446) and the test set (AUC = 0.809 vs. 0.735, *p* = 0.295), although the difference was statistically insignificant. For PR prediction, the multi-task model outperformed the single-task model in the validation set (AUC = 0.748 vs. 0.689, *p* = 0.481) and the test set (AUC = 0.767 vs. 0.688, *p* = 0.248). Similarly, for HER2 prediction, the multi-task model performed much better than the single-task model in the validation set (AUC = 0.712 vs. 0.571, *p* = 0.182) and the test set (AUC = 0.697 vs. 0.626, *p* = 0.394). However, according to DeLong’s test, no significant difference was observed between pairwise models for each biomarker prediction in either the validation or test set.

As for the statistical power, a sample size of 60 patients (46 with positive and 14 with negative) was required for ER in the test set, 39 patients (27 with positive and 12 with negative) for PR prediction, and 68 patients (37 with positive and 29 with negative) for HER2 prediction. Therefore, the sample sizes in this study (88 in the test set) were adequate to detect the true AUCs of 0.735 (for predicted ER), 0.767 (for predicted PR), and 0.697 (for predicted HER2) different from 0.500 with 80% power in the test set.

### Overall prediction performance of different models

The performance of the single-task and multi-task models in predicting overall outcomes is presented in Table 4. The results demonstrate that the multi-task model outperforms the single-task model in all multi-label evaluations, both in the validation and test sets. Specifically, in the test set, the multi-task model achieved an macro AUC of 0.733, macro ACC of 0.588, macro Precision of 0.804, macro Recall of 0.694, and macro F1 score of 0.738, whereas the corresponding figures for the single-task model were 0.708, 0.535, 0.792, 0.634, and 0.693, respectively. In the validation set, the multi-task model also kept better performance than single-task in a small margin (macro AUC = 0.734 vs. 0.691, macro ACC = 0.539 vs. 0.529, macro Precision = 0.798 vs. 0.759, macro Recall = 0.643 vs. 0.629, macro F1 score = 0.697 vs. 0.683).

**Table 4:** Overall prediction performance of single-task model, multi-task model

	macro AUC (95% CI)	macro ACC (95% CI)	macro Precision (95% CI)	macro Recall (95% CI)	macro F1 score (95% CI)
**Validation set (*n*** = **60)**					
Multi-task model	0.734 (0.622–0.846)	0.539 (0.413–0.665)	0.798 (0.696–0.900)	0.643 (0.522–0.746)	0.697 (0.581–0.813)
Single-task model	0.691 (0.574–0.808)	0.529 (0.403–0.655)	0.759 (0.651–0.867)	0.629 (0.507–0.751)	0.683 (0.565–0.801)
**Test set (*n*** = **88)**					
Multi-task model	0.733 (0.641–0.825)	0.588 (0.485–0.691)	0.804 (0.721–0.887)	0.694 (0.598–0.790)	0.738 (0.646–0.830)
Single-task model	0.708 (0.613–0.803)	0.535 (0.429–0.637)	0.792 (0.707–0.877)	0.634 (0.533–0.735)	0.693 (0.597–0.783)

*N* represented the number of involved patients in each dataset

Furthermore, we conducted a stratification in the test set based on lesion sizes, using a criterion inspired by previous studies [25, 26]: lesions with a diameter greater than 15 mm were compared against those smaller than 15 mm. The results, shown in Supplementary Table 1, indicate that the proposed multi-task method consistently outperforms the single-task model for the PR/HER2 individual evaluation and shows better overall performance on both small and large lesions (macro AUC = 0.752 vs. 0.691 for the small lesions and 0.723 vs. 0.715 for the large lesions).

### Model visualization

**Fig. 5:**
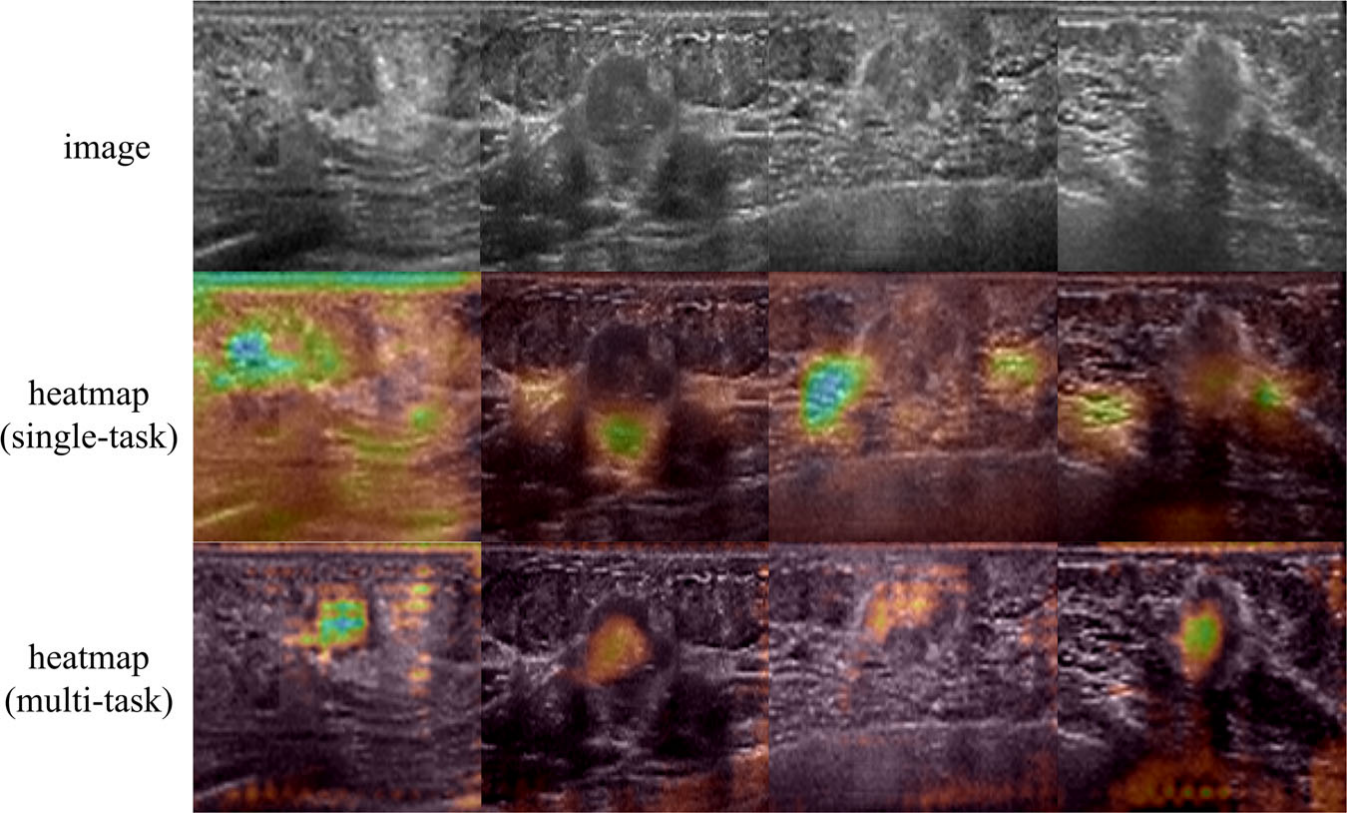
Representative 3DWBUS images and the corresponding Grad-CAM + + heatmaps. The blue or red regions represent areas activated by the multi-task model with higher activation, while the purple regions represent those with lower activation

The Grad-CAM + + heatmaps, resulting from the visualization in the test set, were superimposed on the 3DWBUS images, as illustrated in Fig. 5. Notably, both models showed a significant preference for blue or red areas, whereas they paid less attention to the purple regions. Furthermore, the Grad-CAM + + results demonstrated that the multi-task model exhibited an increased focus on the lesion regions in specific samples of the test set.

**Fig. 6:**
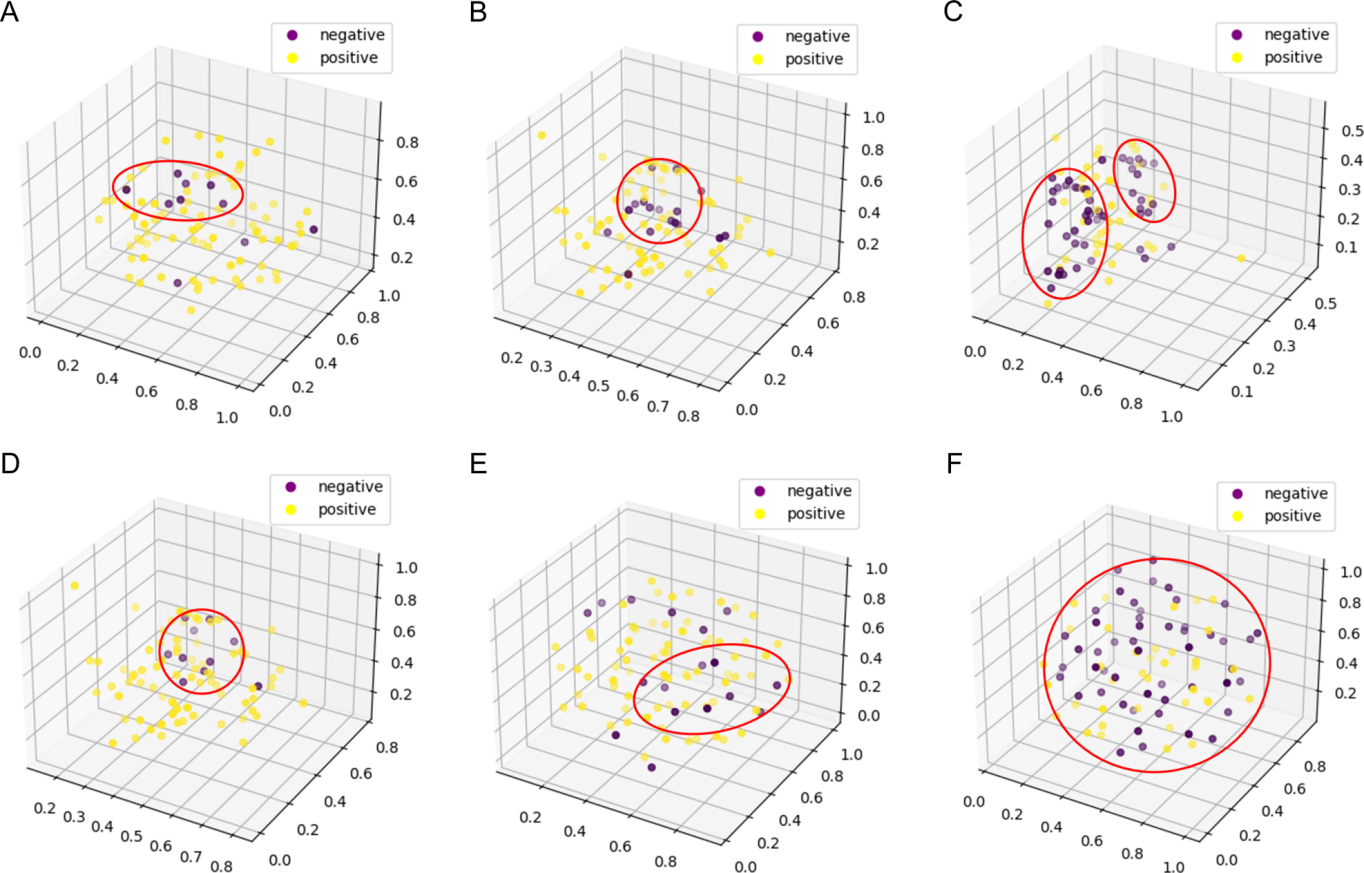
Three-dimensional representation of the distribution of features extracted from the multi-task model for prediction of ER (plot **A**), PR (plot **B**), HER2 (plot **C**). Three-dimensional representation of the distribution of features extracted from the single-task model for prediction of ER (plot **D**), PR (plot **E**), HER2 (plot **F**)

The results of the t-SNE analysis shown in Fig. 6 indicate that, overall, the features extracted by the multi-task model exhibit a greater ability to distinguish between PR and HER2 positive and negative samples compared to the single-tasking model. As shown in Fig. 6B, C, based on the features computed by the multi-task approach, we can clearly identify the red circle that maximally distinguishes as many positive and negative sample categories as possible. As shown in Fig. 6E, F, this is challenging for the single-task approach. In contrast, as shown in Fig. 6A, D, the single-task model exhibits superior discriminatory ability in ER prediction.

## Discussion

We developed and validated both a single-task model and a multi-task model on 3DWBUS images for efficiently predicting the expression of three clinically relevant biomarkers to breast cancer.

Both models based on 3DWBUS images demonstrated satisfactory results for breast cancer biomarker expression prediction (macro AUC > 0.7 in the test set), underscoring the good performance of the deep learning models. Moreover, the multi-task model consistently outperformed the single-task model across various metrics, indicating its superior performance. Specifically, the multi-task model showed improvements across all evaluation metrics for overall prediction. Regarding individual biomarker prediction, the multi-task model achieved higher AUC values for PR and HER2, but a lower AUC value for ER. This indicates a trade-off where the model, while losing some accuracy in ER prediction, gains significantly in predicting PR and HER2, leading to an overall enhancement in performance. Notably, the image features extracted by the multi-task model exhibited clear differentiation for PR and HER2 between positive and negative samples, as confirmed by t-SNE analysis. Conversely, the single-task model exhibited superior t-SNE visualization for ER prediction. These findings partially account for the performance disparity between the two methods. Moreover, the multi-task model improves explainability, especially when considering the segmentation task. The Grad-CAM + + heatmap results revealed that the multi-task model specifically focused on the breast cancer tumor. It extracted lesion area features for the entire tumor, instead of other breast structures, unlike the single-task model. This feature contributes to a better understanding and interpretability of how the multi-task model functions, which further proves our hypothesis.

Indeed, the multi-task model outperformed the single-task model across various metrics, regardless of whether it is individual or overall prediction. This can be attributed to the fact that the single-task deep learning model extracted high-dimensional features from 3DWBUS without any constraints, and hence, has selected some redundant features as shown in Fig. 5. As the study by Feng et al [27] revealed, lesions with 3DWBUS imaging features such as the retraction phenomenon strongly correlate with the molecular subtypes. The single-task model failed to extract sufficient lesion-related features, thereby hindering accurate prediction. In contrast, the multi-task model introduced an explicit segmentation task that compelled the classification branch to extract more relevant information about the lesion [21]. This aligns with the underlying mechanism of multi-task learning, as concluded by Zhang et al [28], and provides a plausible explanation for the superior performance of the multi-task model.

Several studies have explored the use of AI technology to predict breast cancer biomarkers’ expression. Nevertheless, most of these algorithms [7, 29–32] were developed using whole slide images scanned from the histopathological slides. However, the pathological images are much more local representations of the lesion, from the biopsy alone and may be biased by the sample location etc. Furthremore, pathology examinations are invasive and patient unfriendly. In contrast, our study opted to use comprehensive and noninvasive 3DWBUS images to predict biomarkers, and the single-task and multi-task models demonstrated a similar prediction performance to previous pathology-based studies [3].

Similar to our study, some studies have utilized US images to develop noninvasive methods. Zhang et al [8, 9] utilized multi-modality mammography and US for accurate prediction. Although our multi-task model’s performance was not as high as Zhang’s, it is possible that our single-modality (B-mode only) approach constrained the feature source and limited the performance. Nevertheless, our multi-task approach demonstrated comparable performance, showcasing its ability to effectively leverage the information contained within a single-modality image for precise predictions. Moreover, our approach directly predicted the expression of biomarkers rather than specific tumor typing as in the research mentioned above, which is challenging but clinically useful as it enabled clinicians to identify the expressed biomarkers directly and facilitated targeted drug screening. Although this approach degraded our models’ performance, it enhanced interpretability and promoted acceptance by physicians. Furthermore, our method encompasses all three biomarkers in contrast to previous studies [31–33] that focused solely on predicting a single biomarker—either ER, PR, or HER2. This comprehensive approach aligns more closely with clinical requirements, offering a more holistic view for diagnostic purposes [34, 35].

Additionally, many AI-based methods have been developed to predict the molecular subtypes of breast cancer based on MRI [36–38]. Ha et al [39] (*n* = 216, *n* represents the sample size), Zhang et al [40] (*n* = 244) and Sun et al [41] (*n* = 266) have each developed MRI-based deep learning models for this purpose. However, the relatively small dataset sizes in these studies potentially limit the performance of their models. Moreover, the use of MRI is often constrained by various factors, including high examination costs, limited scanner availability, the necessity for contrast agent injection, and extended waiting times, etc. Consequently, MRI images may not be readily accessible to every patient. In contrast, utilizing a more substantial dataset (*n* = 388), our study demonstrates superior performance over the methods above, capitalizing on 3DWBUS images’ advantages such as standardization, lower costs, and reduced examination time.

We acknowledge several limitations in this study. First, it should be noted that this is a retrospective study. To more robustly ascertain the efficacy of the proposed method, conducting additional prospective experiments is imperative. Second, the study was performed at a single center. While our results were reliable and feasible based on sample size calculations, future studies with larger sample sizes from multiple centers will be necessary to validate our prediction performances. Third, the HER2 subgroup had a relatively small sample size due to the lower frequency of this type of breast cancer in clinical reality. This category imbalance problem certainly affects the performance of HER2 prediction (Table 3). In future studies, it will be necessary to include more data on HER2 subtype to achieve a balanced predictive performance across multiple molecular typings. Fourth, the 3DWBUS images used in this study were obtained from only one US device. Hence, the generalizability of the models still requires further validation. A multi-device validation process is essential to ensure broader applicability and reliability of these models. Finally, although our multi-task model showed a better heatmap than the single-task model, it was still challenging to interpret the biological meaning of the extracted imaging features and predictions made by the deep learning method. Investigating the biological significance of these imaging features requires further research [42].

Recently, various single-task deep learning methods have shown promise in different WBUS applications, such as predicting lymph node metastases for local staging [43], guiding clinical management [44] and evaluating treatment response [45]. Our research focuses on breast cancer subtype prediction, and our multi-task model has demonstrated significant potential in this area, achieving better results compared to single-task models. We believe that our model has the potential to be transferred to other applications, thereby achieving better performance in these areas. In the future, we will further refine our model and validate its applicability in different clinical scenarios, ultimately facilitating its implementation in routine clinical practice.

In conclusion, our study demonstrates that both single-task and multi-task models can accurately predict biomarkers’ expression from breast cancer 3DWBUS images, with the multi-task model exhibiting superior performance and enhanced explainability. Utilizing Grad-CAM + + technology, the heatmap generated by the multi-task model showed a more concentrated focus on the lesion region. Although further improvement and validation are necessary before automated breast cancer biomarker prediction models can be integrated into clinical workflows, their notable accuracy and interpretability position these methods as valuable supplements to current clinical practices. Their initial utility may extend to research and quality control applications. For instance, biomarker-based selection or triage of patients in large clinical trials could significantly streamline therapy development pipelines. In addition, this approach could facilitate the identification of more informative tumor regions for biomarker evaluation, as indicated by heatmaps generated using Grad-CAM + +. Last, this research lays the groundwork for future studies aimed at comparing clinical workflows with and without the incorporation of this advanced machine-learning framework.

## Supplementary information

ELECTRONIC SUPPLEMENTARY MATERIAL

## Data Availability

The data analyzed during the current study are available from the corresponding author on reasonable request.

## Abbreviations

3DWBUS: 3D whole breast ultrasound system
ACC: Accuracy
AI: Artificial intelligence
AUC: Area under the receiver operator characteristic curve
ER: Estrogen receptor
HER2: Human epidermal growth factor receptor 2
MRI: Magnetic Resonance Imaging
PR: Progesterone receptor
ROC: Receiver operating characteristic
SEN: Sensitivity
SPEC: Specificity
t-SNE: t-distributed stochastic neighbor embedding algorithm
US: Ultrasound
