# Supplementary material for: Explainable breast cancer molecular expression prediction using multi-task deep-learning based on 3D whole breast ultrasound
Source: Insights Imaging. 2024 Sep 19;15:227. doi: 10.1186/s13244-024-01810-9 (PMC11424596; doi:10.1186/s13244-024-01810-9)
Supplement: Supplementary file 1 — ELECTRONIC SUPPLEMENTARY MATERIAL [file 13244_2024_1810_MOESM1_ESM.pdf]

Explainable breast cancer molecular expression prediction using multi-task deep-learning based on 3D Whole Breast Ultrasound

ELECTRONIC SUPPLEMENTARY MATERIAL

Data pre-processing

Normalization. As the 3D WBUS images were different from their voxels spacing, which might be an adverse factor to modeling, we applied resampling to the images into the spacing of 0.5 mm×0.5 mm×0.5 mm by trilinear interpolation, followed by transforming the values to [0, 1] by min-max normalization:

$$I' = \frac{I - min\,(I)}{max\,(I) - min(I)}$$

where I represents the original image, I' represents the normalized image, $max\,(I)$ represents the maximum value of the original image, and $min\,(I)$ represents the minimum value of the original image.

Cropping. We expanded the boundaries of the volumes of interest drawn by radiologists with 30 to 50 voxels, constructed a new bounding box combing the tumor and cropped the volumes from the original ABUS images.

Resizing. The input image of the convolutional neural network needed to be the same size so we resampled the cropped 3D images to 128×128×128 voxels.

Supplementary Table 1 Results of stratified analysis based on 15mm of tumor size

Test set (n = 88)	AUC(95% CI)	ACC(95% CI)	SEN(95% CI)	SPEC(95% CI)	p*
<15mm (n = 21)					
ER					
Multi-task model	0.768(0.489-0.988)	0.667(0.465-0.869)	0.600(0.390-0.810)	0.833(0.673-0.993)	0.776
Single-task model	0.792(0.502-0.995)	0.714(0.521-0.907)	0.667(0.465-0.869)	0.833(0.673-0.993)	
PR					
Multi-task model	0.778(0.493-0.992)	0.762(0.580-0.944)	0.733(0.544-0.922)	0.833(0.673-0.993)	0.324
Single-task model	0.668(0.402-0.925)	0.619(0.411-0.827)	0.600(0.390-0.810)	0.667(0.465-0.869)	
HER2					
Multi-task model	0.711(0.457-0.953)	0.714(0.521-0.907)	0.600(0.616-0.806)	0.727(0.498-0.702)	0.313
Single-task model	0.613(0.389-0.856)	0.619(0.411-0.827)	0.600(0.390-0.810)	0.636(0.430-0.842)	
>15mm (n = 67)					
ER					
Multi-task model	0.722(0.525-0.919)	0.679(0.567-0.791)	0.660(0.547-0.773)	0.714(0.606-0.822)	0.229
Single-task model	0.811(0.629-0.989)	0.687(0.576-0.798)	0.623(0.507-0.739)	0.929(0.868-0.990)	
PR					
Multi-task model	0.759(0.562-0.935)	0.739(0.634-0.844)	0.702(0.592-0.812)	0.800(0.704-0.896)	0.536
Single-task model	0.699(0.503-0.901)	0.644(0.529-0.759)	0.617(0.501-0.733)	0.800(0.704-0.896)	
HER2					
Multi-task model	0.689(0.493-0.882)	0.642(0.527-0.757)	0.750(0.646-0.854)	0.590(0.472-0.708)	0.589

Single-task model	0.634(0.435-0.836)	0.627(0.511-0.743)	0.679(0.567-0.791)	0.590(0.472-0.708)
